# Predicting Housekeeping Genes Based on Fourier Analysis

**DOI:** 10.1371/journal.pone.0021012

**Published:** 2011-06-08

**Authors:** Bo Dong, Peng Zhang, Xiaowei Chen, Li Liu, Yunfei Wang, Shunmin He, Runsheng Chen

**Affiliations:** 1 Bioinformatics Laboratory and National Laboratory of Biomacromolecules, Institute of Biophysics, Chinese Academy of Sciences, Beijing, People's Republic of China; 2 Key Laboratory of the Zoological Systematics and Evolution, Institute of Zoology, Chinese Academy of Sciences, Beijing, People's Republic of China; 3 Graduate School of the Chinese Academy of Sciences, Beijing, People's Republic of China; Duke University, United States of America

## Abstract

Housekeeping genes (HKGs) generally have fundamental functions in basic biochemical processes in organisms, and usually have relatively steady expression levels across various tissues. They play an important role in the normalization of microarray technology. Using Fourier analysis we transformed gene expression time-series from a Hela cell cycle gene expression dataset into Fourier spectra, and designed an effective computational method for discriminating between HKGs and non-HKGs using the support vector machine (SVM) supervised learning algorithm which can extract significant features of the spectra, providing a basis for identifying specific gene expression patterns. Using our method we identified 510 human HKGs, and then validated them by comparison with two independent sets of tissue expression profiles. Results showed that our predicted HKG set is more reliable than three previously identified sets of HKGs.

## Introduction

A housekeeping gene (HKG) is typically a constitutive gene which is required for the maintenance of basic cellular functions, and generally has a steady expression level across various tissues through all phases of cell development irrespective of environmental conditions. This makes HKGs excellent controls for the normalization of Gene Chip technology, and allows the sample quality and consistency of sample quantity on chips to be assessed [1]. The development of high-throughput gene analysis has enabled more precise investigation of gene expression patterns during various cell development phases and has identified some putative characteristics of HKGs. Using the Affymetrix HuGeneFL chip, Warrington et al. [2] and Hsiao et al. [3] identified 533 and 451 HKGs, respectively, from about 7000 genes by sampling 11 and 19 different tissues. Eisenberg et al. [4] subsequently identified a set of HKGs containing 575 genes using data from a more advanced Affymetrix U95A platform based on 47 tissue samples. However, these three HKG sets contain a total of 963 genes, but only have 158 genes in common. This lack of consistency between datasets implies that there exist a number of false positives and negatives within existing HKG sets, and is due to a lack of agreement on the defining characteristics of HKGs. In addition, high levels of background noise and reproducibility problems are difficult to avoid in microarray experiments.

Eisenberg et al. [4] identified several characteristics of HKGs. They proposed that HKGs usually have shorter introns, UTRs and coding sequences, reasoning that a more compact gene structure should facilitate more efficient transcription, particularly in the case of ubiquitously expressed HKGs. A more compact gene structure is consistent with the stable expression of HKGs across tissues and developmental stages since, in comparison with tissue-specific genes, HKGs likely do not require complex transcriptional control. Vinogradov et al. [5] proposed that the intergenic regions between HKGs are also shorter. However, results reported by Zhu et al. [6] on comparisons of ESTs from HKGs and tissue-specific genes suggest that HKGs do not have a compact gene structure, creating some confusion on how the characteristics of HKGs should be defined. Research on HKG gene sequences includes analysis of the frequency of simple sequence repeats (SSR) in the 5′-UTRs [7], content of repetitive sequences [8], and CG-abundance [9]. Farre et al and Zhang et al worked on the evolution and conservation of the gene sequence or the upstream sequence of HKGs and tissue specific genes.

However, even if there was strong agreement on these defining features of HKGs, these characteristics by nature are not powerful or sufficient enough to decisively discriminate between HKG and non-HKG genes. Thus, at present there is no effectual algorithm for reliably predicting HKGs.

Existence of natural bio-rhythms implies that HKGs, which are constitutively expressed in all cell types and phases, may have certain expression frequency patterns. These spectral features can be extracted using harmonic analysis of gene expression time series and used for predicting HKGs. Here, in order to develop a method for discriminating HKGs on the basis of expression features, we introduced discrete Fourier transform of finite length time series [10] into gene expression data analysis, and classified the spectral patterns obtained using machine learning methods. We then constructed an HKG prediction process and obtained and verified a set of 510 HKGs.

## Methods

### Selection of gene expression time-series data

Fourier analysis requires data with a long series length and high sampling density. Unfortunately, this requirement is much too rigorous for most standard biochemical experiments. In addition, the length of a time series is not easily extended, for example, cells synchronized by serum starvation gradually lose their phase coincidence after several cycles of cell division, thus causing the Gauss distribution to broaden. If cells continue to divide in an unsynchronized manner, cell cycle phases will totally vanish and information from an extended time series will be meaningless.

To satisfy these requirements, we selected a set of human Hela cell gene expression time-series, each with 47 sampling points which were spaced 1 hour apart, covering three cell cycles [11], [12] (http://genome-www.stanford.edu/Human-CellCycle/HeLa/).

### Pre-processing of time-series data

**Figure 1:**
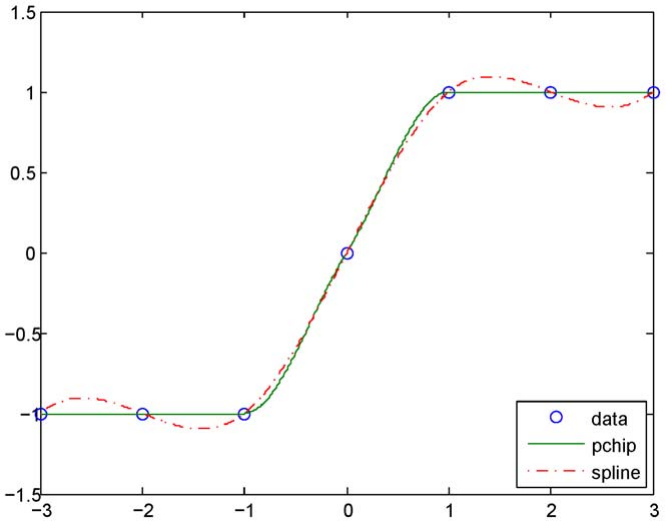
Hermite interpolation. Hermite interpolation (pchip) maintains the shape of the data better than cubic spline interpolation.

It is almost inevitable that there will be some missing data points in a gene expression time series. Here, we eliminated series which had successive missing points or three or more separated missing points, since non-uniform sampling is problematic in Fourier analysis. Series that had one or two separated missing points were interpolated with piecewise cubic Hermite interpolation, a relatively conservative algorithm which does not overshoot and introduces less oscillation (Figure 1), since the expression data were not smooth. In this way we constructed a dataset which contained 32,786 uniform sampling expression time series covering 15,261 genes.

**Figure 2:**
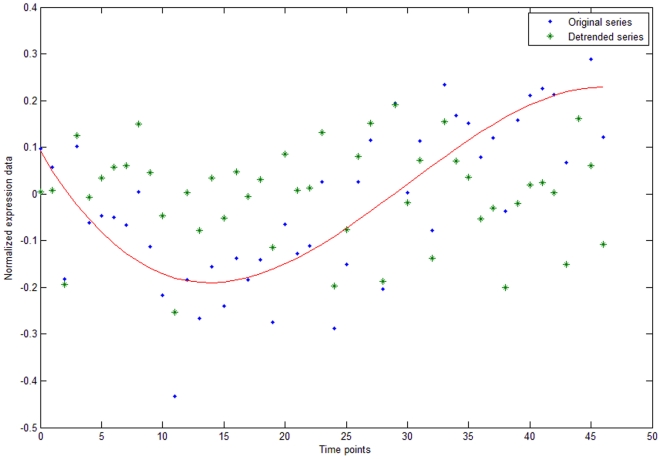
The effect of removing non-periodic trends from the data. The variation trend for the original time series is shown by a red line. After elimination of this trend the data will be at least first order stationary.

Generally speaking, these time series were not stationary, i.e. their mean values varied with time. In order to uncover the periodical components of the data by Fourier analysis, we eliminated trends and seasonal components using the least squares method with five variation bases, transforming the time series into at least a first order stationary series. The principle of variation used to fit the series with variation bases was to minimize the grand total square errors (Figure 2).

Taking a series with p time points as a vector with p components, 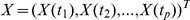, we can approximate the vector with q base functions 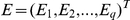. The approximate error 
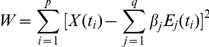
 is minimized when the linear equations 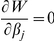 are satisfied.

Here we chose five base functions 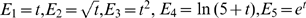.

**Figure 3:**
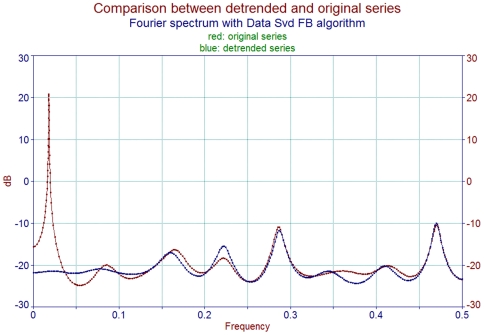
Fourier spectrum of the same time series as in the **Figure 2**. Meaningless long period terms were filtered out after the variation process. The main periodical components in the original series are preserved in the processed time series.

The logarithm term was derived from the Frobenius method for second order differential equations which implies that the gene expression time series were continuous and did not contain singularities within the time intervals we concentrated on. Frequency analysis before and after data pre-processing showed the maintenance and enhancement of periodical components in the residual series (Figure 3).

### Interpretation of HKGs

**Figure 4:**
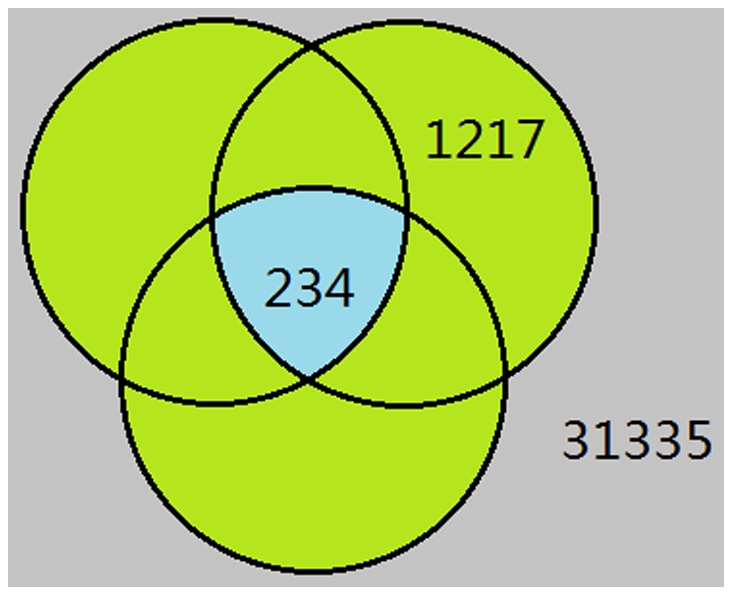
Distribution of the probe sets of genes in the three published HKG datasets. The cyan area in the center represents 234 probes corresponded to genes common to all three HKG sets (Standard HKGs). The green area represents 1217 probes corresponded to genes only in one or two HKG sets (Putative HKGs). The outside grey area represents probes corresponded to other genes not present in any of the HKG sets (non-HKGs).

Warrington [2], Hsiao [3] and Eisenberg [4] each reported sets of HKGs based on the analysis of tissue microarray experiments. In the Hela cell expression data used here, of the 32,786 effective time series, 234 series corresponded to 158 genes which were common to the above published HKG sets, 1217 series corresponded to the 805 genes which were found in only one or two of the published HKG sets, and 31,335 series corresponded to the 14,297 genes which were not present in any of the published HKG sets (Figure 4). We defined these three collections of genes as Standard HKGs, Putative HKGs and non-HKGs, respectively.

### Identification and extraction of the features of HKG spectra

Discrete Fourier transform (DFT) was first applied to time series that had been made stationary in order to enhance the gene expression frequency components of the spectrum. As the time series all contain 47 time points, each separated by 1 hour intervals, we obtained 24 terms from the frequency spectra obtained by applying DFT. The frequency components could be obtained by the formula:
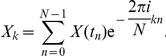


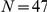
 is the length of each time series. The complex numbers 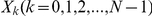 are the Fourier spectrum with frequency 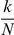 cycles per sampling point and 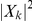 are the power spectrum. For expression data are real numbers, the first 23 frequency terms are conjugate to the last 23 terms, i.e. 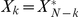. Thus there are only 24 independent components. We used the power spectrum of all these 24 frequency components as our SVM features.

In order to test whether the frequency components of the time series obtained were characteristic features which could be used to distinguish HKGs from non-HKGs, we used a supervised statistical learning method. Generally speaking, whether an HKG expression spectrum has frequency characteristics or not is best determined using Support Vector Machine (SVM). The SVM performed classification by constructing an hyperplane that optimally separates the data into two categories of HKGs and non-HKGs. The goal of SVM modeling was to find the optimal hyperplane that separates clusters of time series in such a way that cases of the HKG category are on one side of the plane and cases of the non-HKG category are on the other size of the plane. Libsvm (Chih-Chung Chang and Chih-Jen Lin, LIBSVM: a library for support vector machines, 2001. http://www.csie.ntu.edu.tw/~cjlin/libsvm) was used here to distinguish between the genes, taking the 24 effective frequency components obtained by Fourier transformation as features. The Gaussian radial basis function (RBF) kernel was adopted with penalty parameter 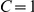 and exponent parameter 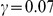. The parameter pair 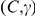 was selected by the commonly used cross-validation.

### Evaluation using tissue expression profiles

Two independent human tissue expression profiles: GSE2361 [13], expression profiling of 36 types of normal human tissues, and GSE1133 [14], mRNA expression pattern of 79 human tissues, were downloaded from NCBI. Probe intensity data was converted to log2 ratios. Intensity data for different probes corresponding to the same genes were then averaged to represent gene expression levels. The standard deviation (SD) and mean value for each gene across tissues were calculated for each dataset. The coefficient of variation (CV; SD/mean) was obtained.

### Gene ontology analysis

A human gene association file (GOC Validation Date: 08/27/2010, CVS Version: Revision: 1.159) was downloaded from the Gene Ontology website [15]. We used the WEGO web server [16] to plot GO results by converting our predicted gene set to the WEGO native format. Only GO level 2 was plotted.

### Gene Conservation Analysis

Human hg18 conservation data for 28 vertebrate genomes (phastCons28way) [17] and a hg18 gene table [18] were downloaded from the UCSC web site. The conservation score of each of the HKG and non-HKG was calculated as the mean value of all exon base phastcons scores of their mRNAs. If a gene had more than one mRNA sequence, all mRNA scores were averaged to give a final score.

For a brief summary of the entire process, please see the part 1 of Text S1.

## Results

### Gene expression frequency spectra can be used as effective characteristics for discriminating HKGs

**Figure 5:**
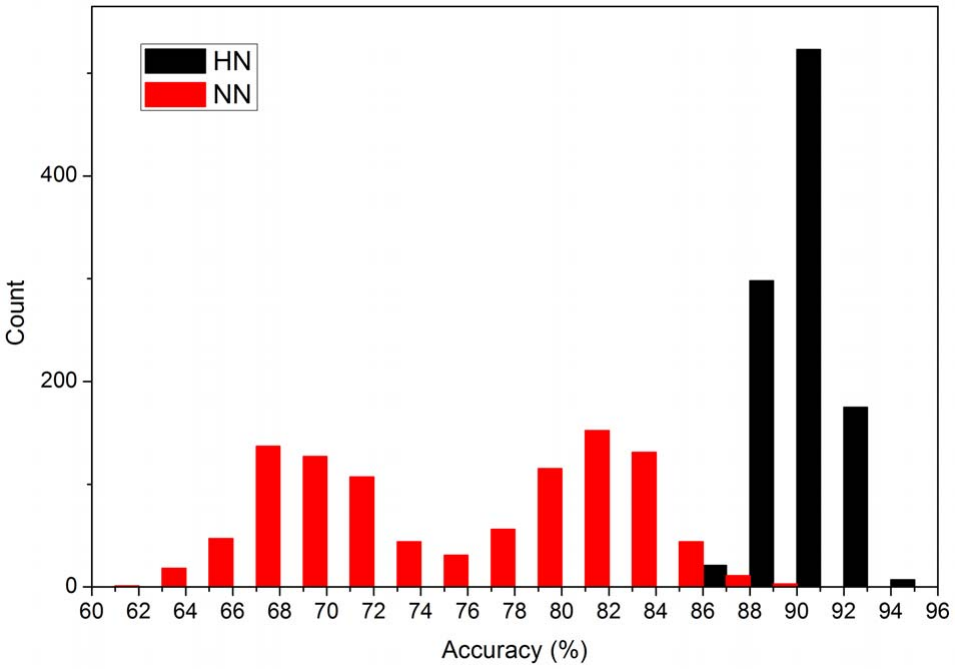
Ability of the HN and NN models to discriminate between HKGs and non-HKGs. The models were replicated a total of 1024 times. SVM can recognize structural differences between the standard HKG and random non-HKG sets better than those of two random non-HKG sets. The accuracy distribution of the NN model has two peaks, suggesting that the non-HKG time series has an intrinsic structure. This is not surprising since the number of non-HKG genes is much larger.

Since HKGs are genes that commonly have stable expression levels at all growth stages in all organisms, there should be conceivable differences in periodic expression features between HKGs and non-HKGs. For this reason we hypothesized that frequency spectrum features could be used to discriminate between HKGs and non-HKGs. Here, we used Whitfield et al.'s Hela cell dataset which contains the time expression series of 41508 probes. Spectral analysis was performed with Discrete Fourier Transform (DFT), and periodical features were identified and extracted from the frequency statistics obtained using SVM (see Methods section). In order to test whether the Fourier spectrum of a gene is a distinct feature of an HKG, we established two classification models based on 24 frequency components obtained with Fourier analysis: the HN model (HKG/non-HKG; true model) and the NN model (non-HKG/non-HKG; control model). In the HN model, the 234 standard HKG probes were used as positive cases and 234 non-HKGs were selected randomly and used as negative cases for SVM. In the NN, or “control model”, 234 random non-HKG probes were used as positive cases and 234 other non-HKG probes were selected randomly and used as negative cases. Figure 5 shows that the efficiency of the NN model in discriminating between HKGs and non-HKGs is markedly lower than that of the HN model. It is thus evident that HKG frequency components have characteristic structures that can be detected by SVM, indicating that the frequency components of gene expression can be used to effectively discriminate between HKGs and non-HKGs. Computational details are given in the Figure S1 and part 2 of the Text S1.

### Prediction of HKGs

**Figure 6:**
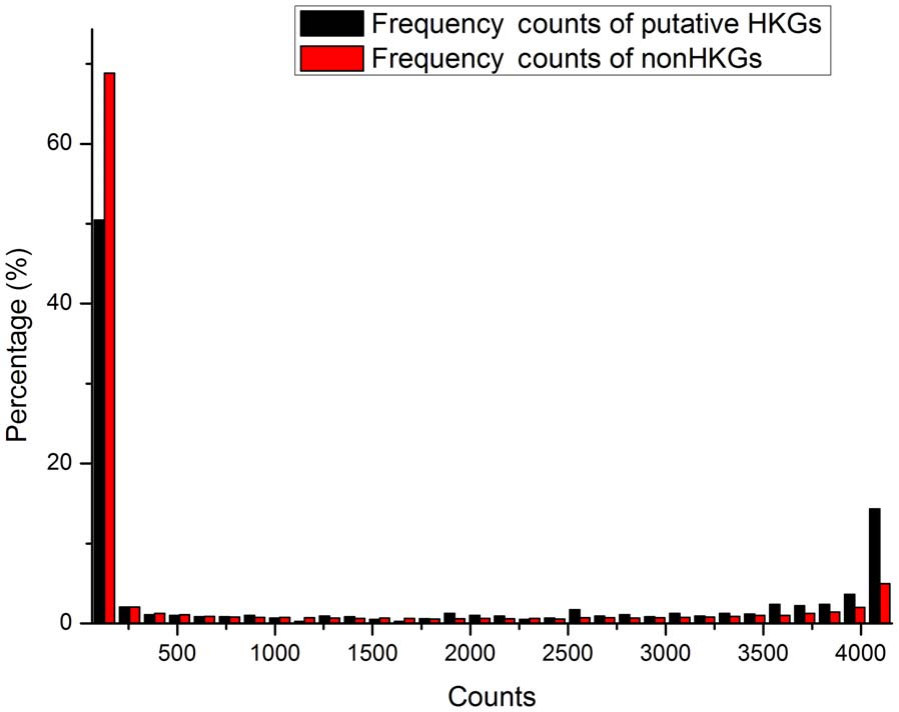
HKG count distribution. 4096 rounds of stochastic SVM classification were performed, each round giving an estimation of whether putative HKGs (black) and non-HKGs (red) were indeed HKGs. The greater the number of counts obtained, the more likely a gene is to be an HKG. The distribution of counts shows that a larger proportion of putative HKGs have a high percentage of counts than non-HKGs, suggesting that there is a larger proportion of HKGs in the putative HKG set than in the non-HKG set.

**Figure 7:**
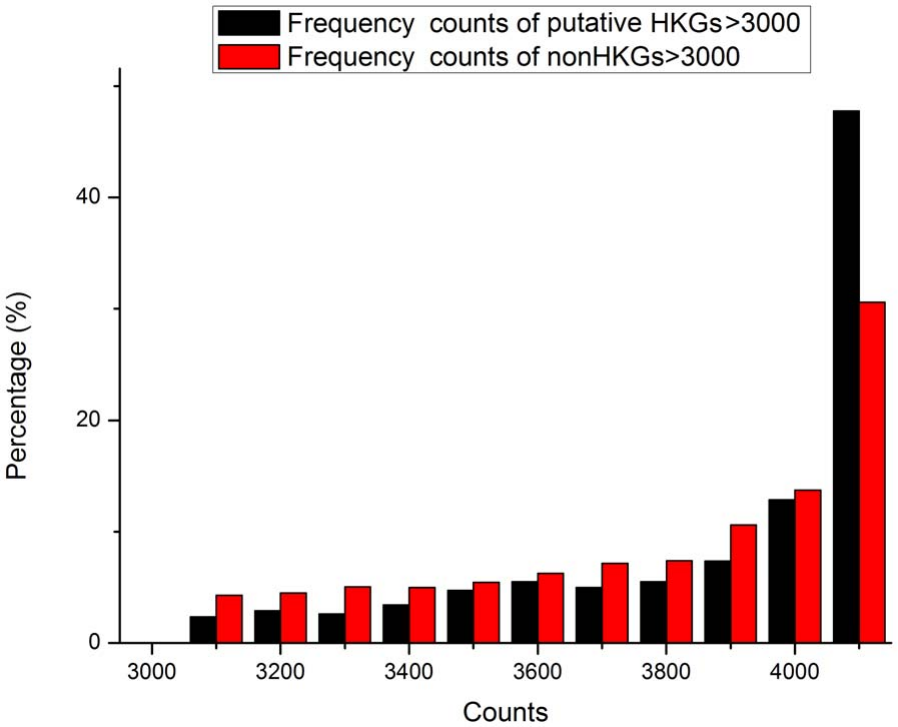
Distribution of counts for probes with more than 3000 counts. We chose 3328 counts as the minimum cut-off point for selection from putative HKG set.

**Figure 8:**
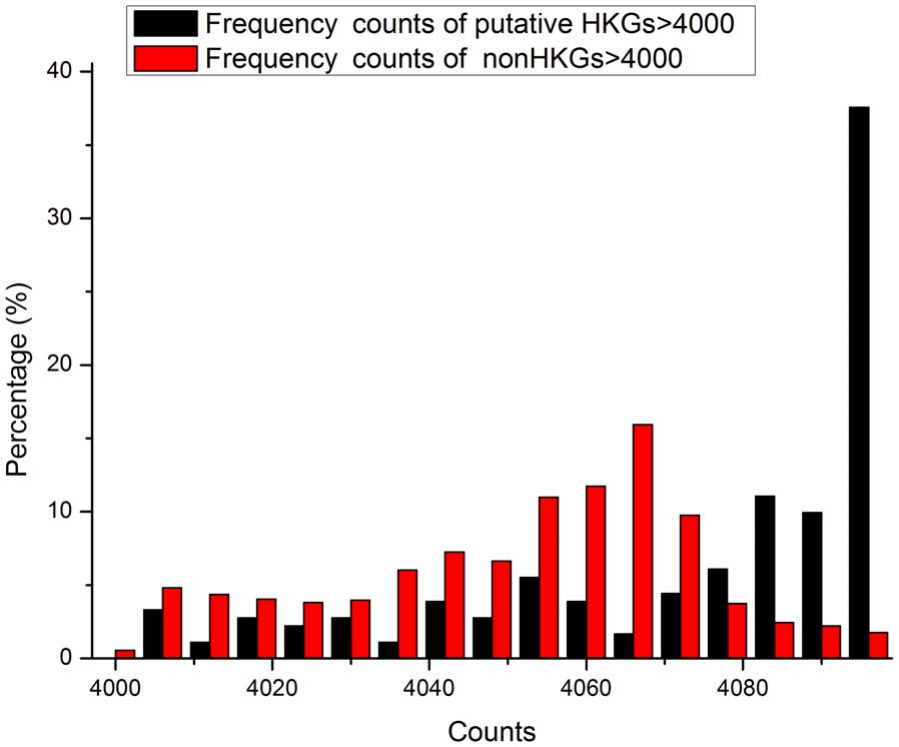
Distribution of counts for probes with more than 4000 counts. Genes in this region are highly likely to be HKGs. It can be seen that the percentage of putative HKGs is much greater than that of the non-HKGs from about 4085 counts, suggesting that 4085 counts is a suitable cut-off criterion for predicting HKGs.

As discussed above, the lower than anticipated overlap between the HKG collections published by Warrington [2], Hsiao [3] and Eisenberg [4] indicates the presence of false positives and false negatives within these datasets. The ability of the HN and NN models to discriminate between HKGs and non-HKGs based on frequency components of gene expression shows that prediction and identification of HKGs is possible. In order to eliminate false positives and false negatives from the set of 805 putative HKG genes in the Hela cell dataset that overlapped with one or two of the published HKG sets, and to further classify and predict HKGs within the Hela cell dataset, we established classification models using the 234 probes for standard HKGs that were common to all three datasets as the positive set for the SVM classification prediction model, and randomly selected 234 probes from the 31,335 non-HKG probes as the negative set. After a single round of prediction, the genes which were classified as HKGs were determined by the identity of the genes contained in the randomly selected negative set, i.e. there was stochastic bias. This bias can be eliminated with a bootstrap approach, i.e. genes classified as HKGs were those which had the highest counts after repeated rounds of model selection and classification. We performed computer simulations using the same method to test whether this method can reliably distinguish different kinds of time series. The simulation details and results are shown in part 4 of Text S1. The simulation results demonstrated that our method can identify different frequency patterns. Figure 6 shows the distribution of counts obtained after 4096 (2^12^) rounds of classification. The proportion of probes that had high counts in the set of putative HKGs that overlapped with one or two of the published HKG sets, was much greater than the proportion of possible non-HKGs, once again showing the validity of frequency features. 299 genes from the 805 putative HKG genes were selected as HKGs in this way, using 3328 counts as the minimum cut-off point for selection (81.25% ballot). 53 genes from the non-HKG set were also selected since each of them was counted as an HKG more than 4085 times (99.73% ballot). Figure 7 and Figure 8 each shows the detailed distribution of counts for probes with more than 3000 and 4000 counts. All 158 standard HKGs common to the three published HKG datasets were selected as HKGs. In total our method predicted 510 HKGs. See Table S1 for detailed gene lists.

### Validation and evaluation of HKG prediction results

**Figure 9:**
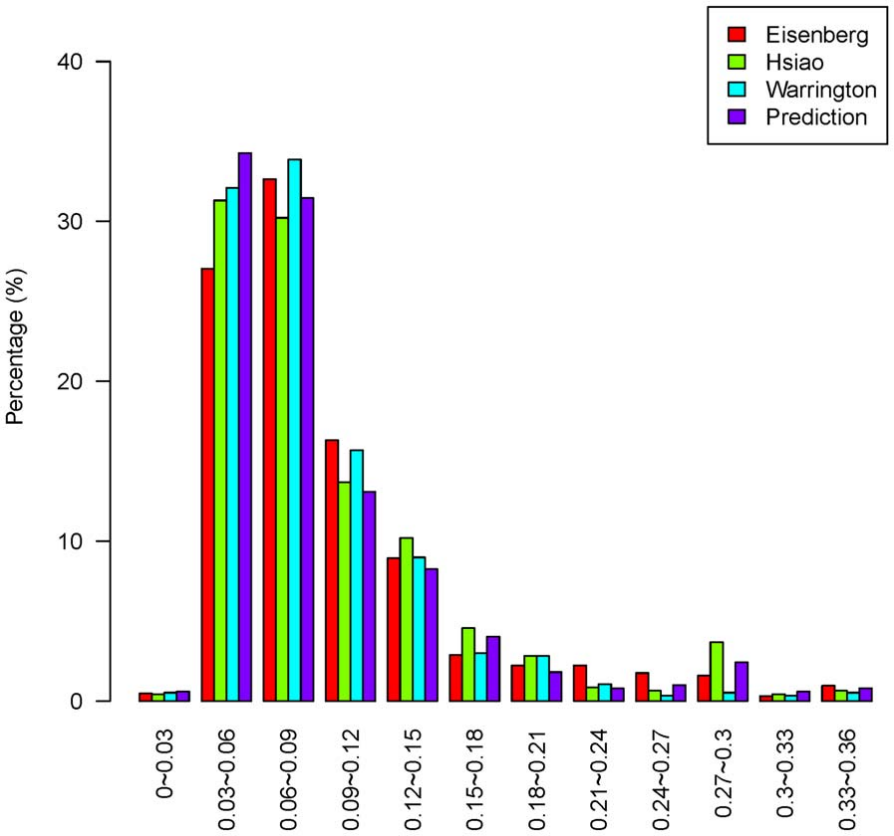
Distribution of CVs (1). Distribution of CVs for the three published HKG datasets and our predicted HKG set using tissue expression data GSE2361 from Ge et al. [13].

**Figure 10:**
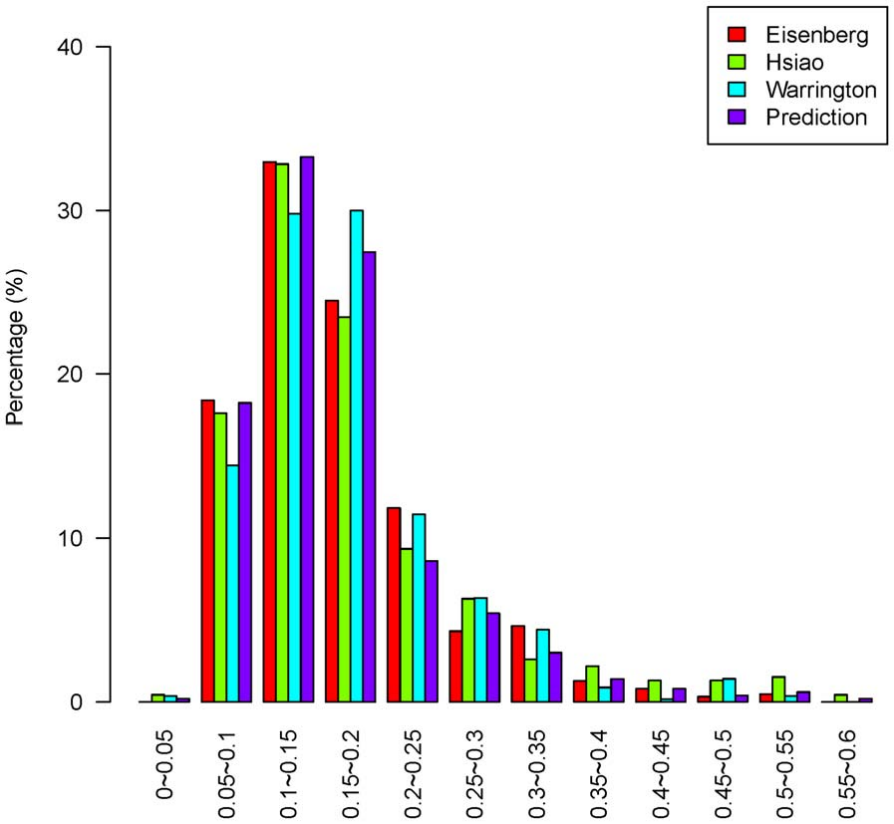
Distribution of CVs (2). Distribution of CVs for the three published HKG datasets and our predicted HKG set using tissue expression data GSE1133 from Su et al. [14].

Our prediction results were evaluated against two sets of tissue expression profiles [13], [14] which were not used in the prediction. These profiles each contained 79 and 36 different tissues. The distribution of the coefficient of variation (CV i.e. SD/mean), a measure of whether a given gene is highly expressed across all tissues and can therefore be considered as an HKG, is shown in Figure 9 and Figure 10 for all the genes in the three published HKG datasets and the 510 predicted HKGs. A comparison of the CVs for our predicted HKGs and all the 15,261 genes in the tissue expression profiles that overlapped with the Hela cell gene expression dataset is shown in Figure S2. CVs of the predicted HKGs tended to be small, suggesting that CV is an appropriate parameter for evaluating HKGs [19].

The median CVs of the two tissue expression profiles are shown in Table 1. The median CV of our predicted set of HKGs is smaller than that of the three published HKG sets, indicating that the genes in our predicted HKG set showed less fluctuation.

**Table 1:** Median CVs.

Median of CV	Eisenberg et. al	Hsiao et. al	Warrington et. al	Predicted HKGs
GSE2361 [13]	0.0785	0.0763	0.0730	0.0720
GSE1133 [14]	0.1462	0.1489	0.1582	0.1462

Our predicted set of HKGs has a smaller median CV than that of the three published HKG sets.

### Gene Ontology Analysis

**Figure 11:**
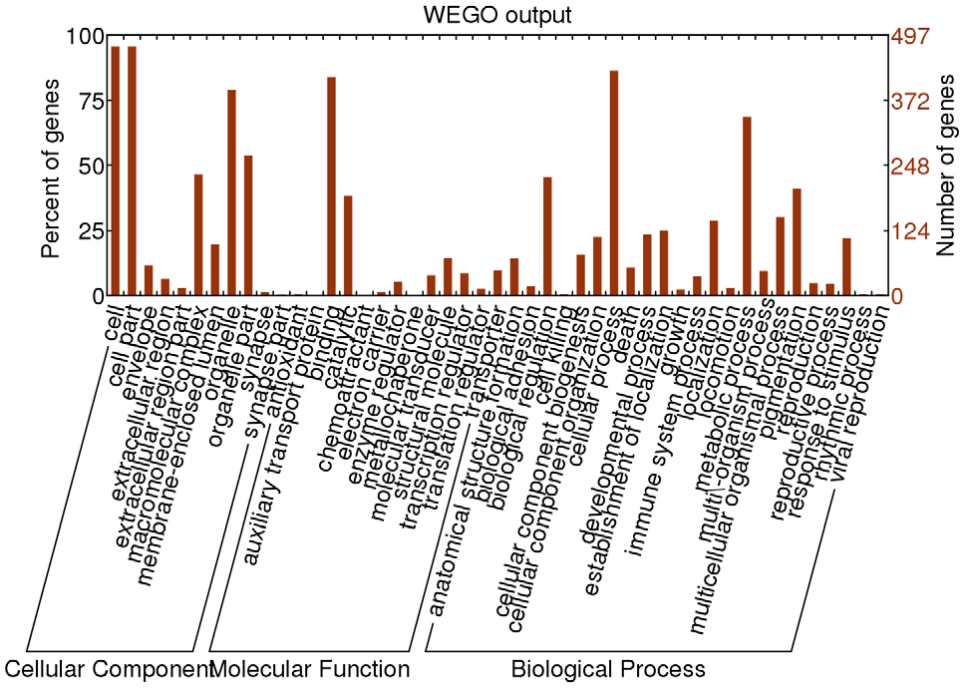
Distribution of gene ontology in our predicted HKG set. GO level 2 is shown. The horizontal axis represents different terms of GO level 2, while the vertical axis represents how many genes or percent of genes in our predicted HKG set belong to each GO term.

We performed a gene ontology analysis to classify the predicted HKGs on the basis of their function (Figure 11). Genes in our predicted HKG set were distributed in several important biological process functional classes including cellular processes, metabolic processes and biological regulation. These terms represent the basal functions that HKGs are responsible for.

### Gene Conservation Analysis

**Figure 12:**
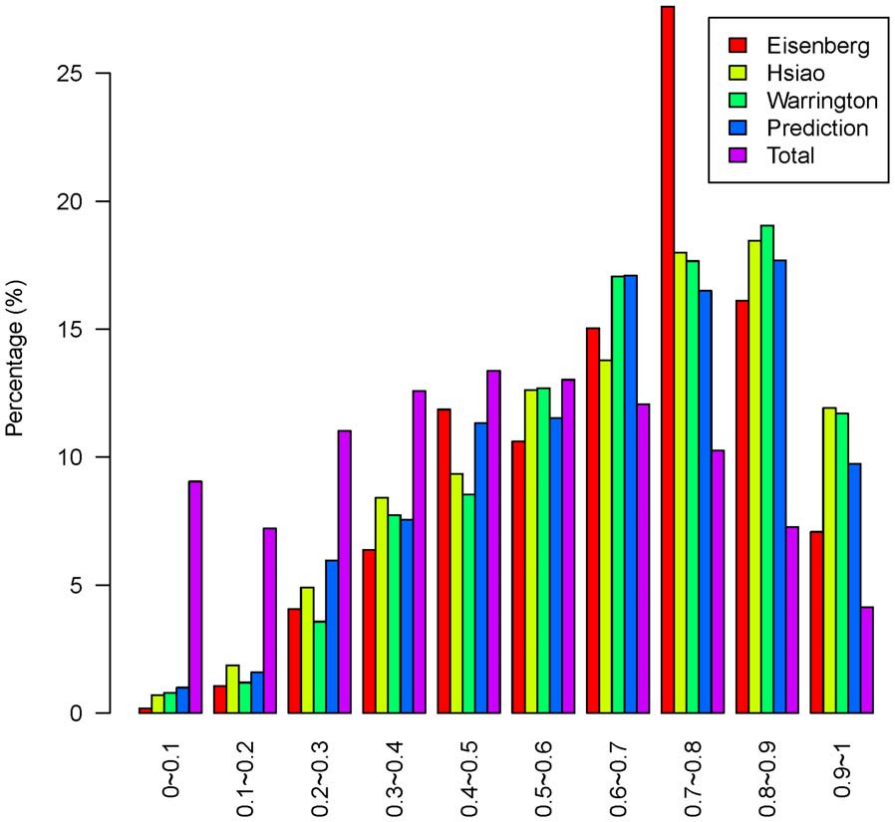
Gene conservation distribution. The horizontal axis represents gene conservation scores.

Figure 12 shows the conservation of different gene sets among 28 species [17]. Genes in all three HKG sets and our predicted HKG set tended to be more conserved than non-HKG genes from the hg18 gene table. The conservation scores of the three HKG sets and our predicted set of HKGs were similar.

## Discussion

### Features of HKGs

HKGs and non-HKGs differ in several statistical quantities such as CG content and SSR density. However, these features are parameters posteriorly-derived from statistical induction, and are therefore not suitable for use in quantitative classification. Such statistical induction is naturally incomplete because sampling processes have unavoidable limitations which tend to result in the choice of different collections of samples being used to address the same problem, and thus in sharply different conclusions. For example, Zhu et al. (2008), and Eisenberg and Levanon have quite different, even opposite, opinions about whether the ESTs of HKGs are compact. Thus, with respect to classification, it is not appropriate to use these statistical quantities as features of high significance and consistency. Classification using our HKG definition and Fourier analysis avoids the use of parameters based on statistical hypotheses. Results from such classifications can be verified by other statistical measures such as differences in tissue expression levels, which are independent of statistical learning and modeling, making the classification more rational.

Some research has shown that expression levels of housekeeping genes may vary depending on experimental conditions [20]. However, unless the cell state is severely disturbed by environmental conditions in the experiments, the most conceivable consequence of this disruption would be uniform upregulation (or downregulation), or gradual increase or decrease in the expression of some genes over the entire length of the experiment. This type of experimental variation will be eliminated by normalization of the data or by pre-processing to remove non-periodic trends. The algorithm used here will be reliable as long as the expression of HKG genes is steady and does not show periodic expression under specific experimental conditions. In other words, the Fourier spectra of two gene expression time-series which seem quite different may be similar, unless they have very different frequency components.

### Different spectral methods

Fourier analysis is an approach which takes advantage of pattern recognition to remove noise from microarray data. A requirement of the DFT method used here is that the data from time series should be steady. The Fourier series expansion is a mathematical description of the physical fact that every linear periodic phenomenon can be expressed by a series of simple harmonic modes. The Fourier coefficient is the weighted mean over the whole time domain, i.e. Fourier analysis shows the properties of an entire time series, instead of being restricted to a small segment. So it is only asymptotic to describe the partial features of time series with it.

Several studies have already extract frequency features from expression time series of cell cycle data using Fourier analysis. The frequency features were further analyzed by functional clustering methods and genes were classified according to different expression patterns across the stages in the cell cycle [21], [22], [23]. de Lichtenberg. et al. [24] constructed an interacting network of cell cycle related proteins by combination of frequency features with physical interacting data. The clustering methods in these works mainly used the most significant frequency components as features. Rustici et al. [23] selected genes with significant power spectrum peaks which were consistent with cell cycle duration. Kim et al. [22] used three main frequency components of Fourier series for clustering, omitting other components. However, housekeeping genes are not related to the cell cycle, and have no dominanting frequencies. We therefore considered all 24 frequency components in our classification. Since SVM is good at distinguishing fuzzy patterns, it is a suitable tool for this type of dichotomy problem. The simulation showed that SVM could recognize various frequency patterns (Text S1 part 4). Our work indicated that housekeeping genes, which are not related to cell cycle, could also be identified from cell cycle data through frequency analysis. However, Cell cycle data are not necessary in the recognization of HKG. We chose these data because they contained the longest available expression time series.

Instant Fourier analysis and wavelet analysis, which consider both time and frequency, can deal with frequencies changing over time. Kim et al. [25] reported a gene clustering work based on wavelet analysis. In fact, when the time series are long enough, wavelet analysis has advantages over traditional Fourier transform for time dependent, non-stationary signals. As the accuracy of microarray data improves and the size of datasets constantly increases, instant Fourier analysis and wavelet analysis will be more often used in biochip data analyses. Using instant Fourier analysis and wavelet analysis, local features within a time series can be identified, such as the response of gene expression to regulating and controlling factors.

### The selection of the threshold

We picked two distinct thresholds for the selection of putative HKG and non-HKG sets. We reasoned that genes in the putative HKG set of the three published datasets are more likely to be HKGs, while those in the non-HKG set are less likely to be HKGs, and thus chose a relatively loose threshold (3328 counts) for genes in the putative HKG set. In fact, a stricter threshold would make the CV of the selected set smaller, but more false negatives would result. We set a much stricter threshold for the non-HKG set (4085 counts), since the relative proportion of suspect HKGs was much greater than that of non-HKGs from about 4085 counts (Figure 8).

### Validation of our predictions via gene function

Some genes from the putative HKG set were rejected by our procedure. For example, TUBB3 was annotated as an HKG in the Eisenberg set, but in fact it is a microtubule element expressed exclusively in neurons, commonly used to identify neurons in nervous tissue. The score for TUBB3 with our prediction method was 2287, below the HKG threshold. In the same way, TUBB scored 0 and was also below the HKG threshold. ILF2 encodes a 45 kDa subunit of NFAT (nuclear factor of activated T-cells), a transcription factor required for T-cell expression of the interleukin 2 gene that is probably only expressed in T-cells and may not be an HKG. CES2 (carboxylesterase 2), expressed in the intestine and liver, is a major intestinal enzyme and functions in intestine drug clearance. It is tissue-specific rather than housekeeping, and was also rejected by our method.

On the other hand, in the non-HKG set, ATG9A scored 4093 and was selected as an HKG. Yamada et al. [26] reported that it is ubiquitously expressed in human adult tissues. The CAPN1 gene which encodes the large subunit of a ubiquitous enzyme, calpain 1, scored 4096 in our study and was also selected as an HKG. UBE2B (score: 4091), the ubiquitin-conjugating enzyme E2B which is required for post-replicative DNA damage repair, is 100% identical to its mouse, rat, and rabbit homologs. UBE2K from the non-HKG set also scored highly (score: 4089) in our procedure. It belongs to the ubiquitin-conjugating enzyme family, too.

Here we have proposed an HKG prediction method using spectral analysis of gene expression time-series data. Our method has proved effectual and we have predicted 510 HKGs using Hela cell cycle data, including 54 genes not present in previously reported HKG sets. Our predicted HKG set was then validated using two independent tissue expression profiles. This method will be further verified when more time series data providing in-depth coverage of a sufficiently long time period become available.

## Supporting Information

Figure S1 **Organization of training and testing sets used by SVM.** Details in the supervised statistical learning process. There are three selected sets used in learning and testing and they are used to test whether the frequency features can be used to recognize HKGs.
(TIF)

Figure S2 **An overall distribution of CVs.** A comparison of the CVs for our predicted HKGs and all the 15,261 genes in the tissue expression profiles that overlapped with the Hela cell gene expression dataset, which suggests that CV is an appropriate parameter for evaluating HKGs.
(TIFF)

Table S1 (XLS)

Text S1 (DOC)
