# Supplementary figures and images for: Predicting Housekeeping Genes Based on Fourier Analysis
Source: PLoS One. 2011 Jun 8;6(6):e21012. doi: 10.1371/journal.pone.0021012 (PMC3110801; doi:10.1371/journal.pone.0021012)

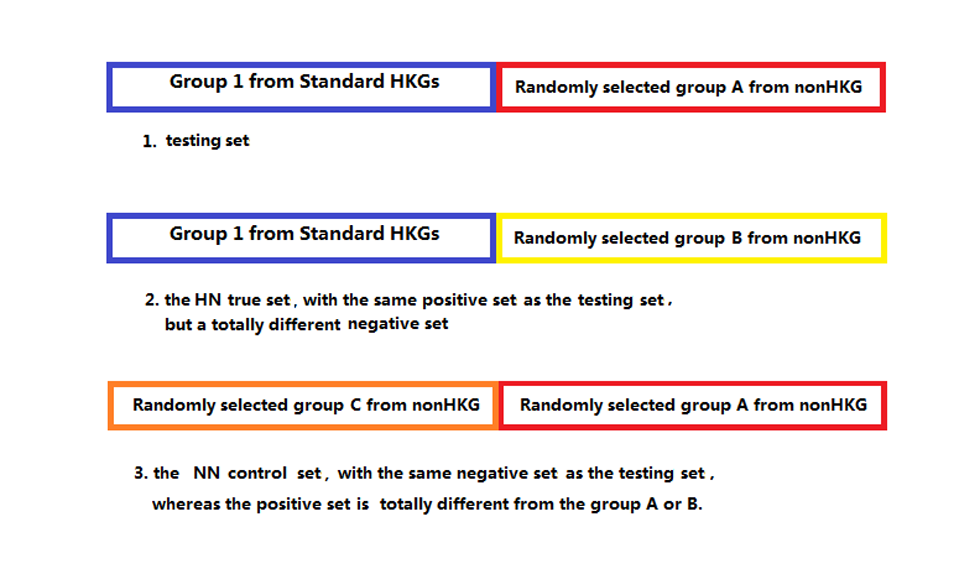

Supplement: Figure S1 — Organization of training and testing sets used by SVM. Details in the supervised statistical learning process. There are three selected sets used in learning and testing and they are used to test whether the frequency features can be used to recognize HKGs. (TIF) [file pone.0021012.s001.tif]

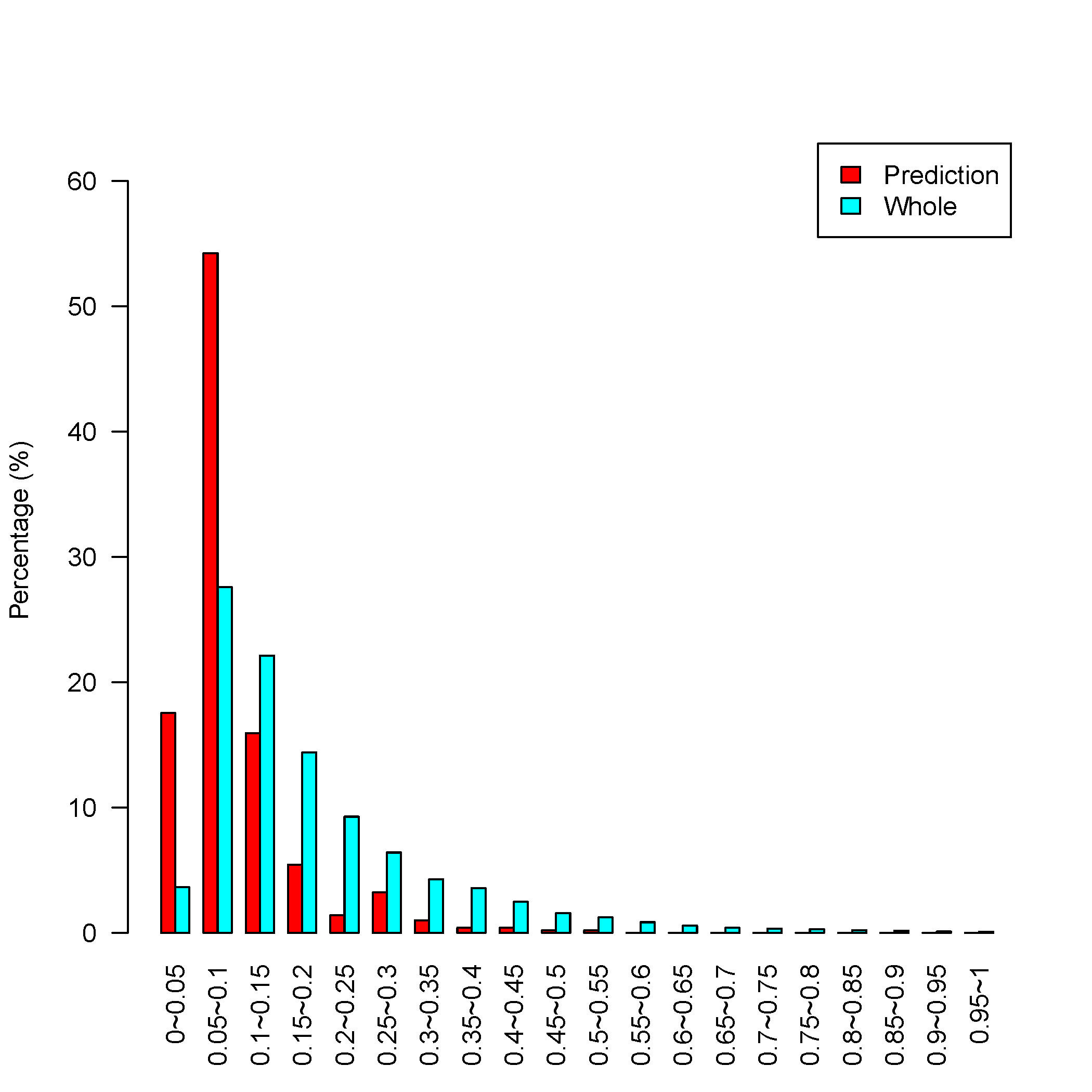

Supplement: Figure S2 — An overall distribution of CVs. A comparison of the CVs for our predicted HKGs and all the 15,261 genes in the tissue expression profiles that overlapped with the Hela cell gene expression dataset, which suggests that CV is an appropriate parameter for evaluating HKGs. (TIFF) [file pone.0021012.s002.tif]
