# Supplementary material for: Predicting Housekeeping Genes Based on Fourier Analysis
Source: PLoS One. 2011 Jun 8;6(6):e21012. doi: 10.1371/journal.pone.0021012 (PMC3110801; doi:10.1371/journal.pone.0021012)
Supplement: Text S1 — (DOC) [file pone.0021012.s004.doc]

# Supporting Information Text S1

1. The Main Process：

Step 1. Selecting and preparing the time series data. The expression time series selected should have high and uniform sampling density, and as long as possible.

Step 2. Preprocessing the data. Use Hermite interpolation to solve the occasional gap of the series. Then eliminate the trend components with method of least squares, in order to make the time series at least the first order stationary.

Step 3. Fourier transform. Transform the time series to the frequency space with Fourier transform. Any appropriate fast algorithm can be used. Here we performed the common discrete Fourier transform to obtain the discrete frequency components of the time series.

Step 4. Validation with statistical learning method. Validate whether the frequency components can be used as sufficient classification features.

Step 5.Classify the time series with the frequency components using support vector machine (SVM) to obtain a single estimation. Then change the training set randomly and do the classification adequate times to get enough estimations. Synthetise all of the estimations and get the final prediction set.

Step 6. Testing the result. Test the predicted set with other data not used in the classification. We use coefficient of variation (CV), which is a definite parameter of HKG, to test the quality of the predicted HKG set.

1. The details about the validation


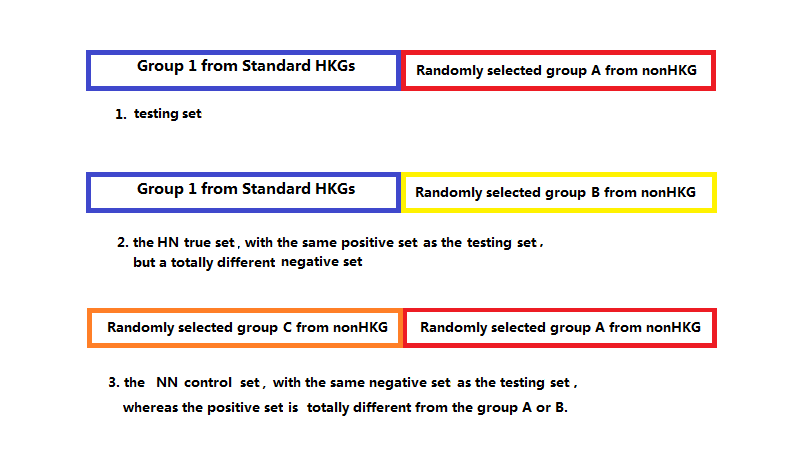


To test whether the HKG set has certain structure which is recognizable by the SVM, we established 3 sets.

The first one is the testing set, used to evaluate SVM models trained by the other two sets below. Its positive part is selected from the set of Standard HKG, while the negative part is randomly selected from the set of non-HKG, called group A in brief.

The second is the HN true training set, used to train the HN true SVM model. Its positive part is just the same as the testing set, whereas the negative part is a very different set randomly selected from the set of non-HKG, called group B. There are no common probes in the group A and B.

The third is the NN control training set, used to train the NN control SVM model. Its positive part is also randomly selected from non-HKGs, but a totally new set different from group A or B, called group C. Its negative part is just the group A, the same as the negative part of the testing set.

In summary, the HN set has the same positive part as the testing set, whereas the NN set has the same negative part as the testing set.

The question is: “Is there any recognizable structure in the HKG set?”

We tested the HN and NN model respectively with the testing set, and found the distinct accuracy difference. Trained with the HN true training set, the SVM model can recognize the positive and negative part of the testing set with accuracy about 90%. However, the NN SVM model can only recognize the positive and negative part of testing set with accuracy about 70% (Figure 5 of the main article). The situations of HN and NN model in training and testing are totally equal. So there must be some kind of pattern structure in the genes of the HKG sets which can be recognized by SVM.

3. Figure S2 shows CV difference between the whole probe set and the prediction set of the tissue expression data GSE2361. The CV coefficient shows the relatively fluctuating according to the expression level so that it is appropriate for judging whether a gene is likely to be an HKG.


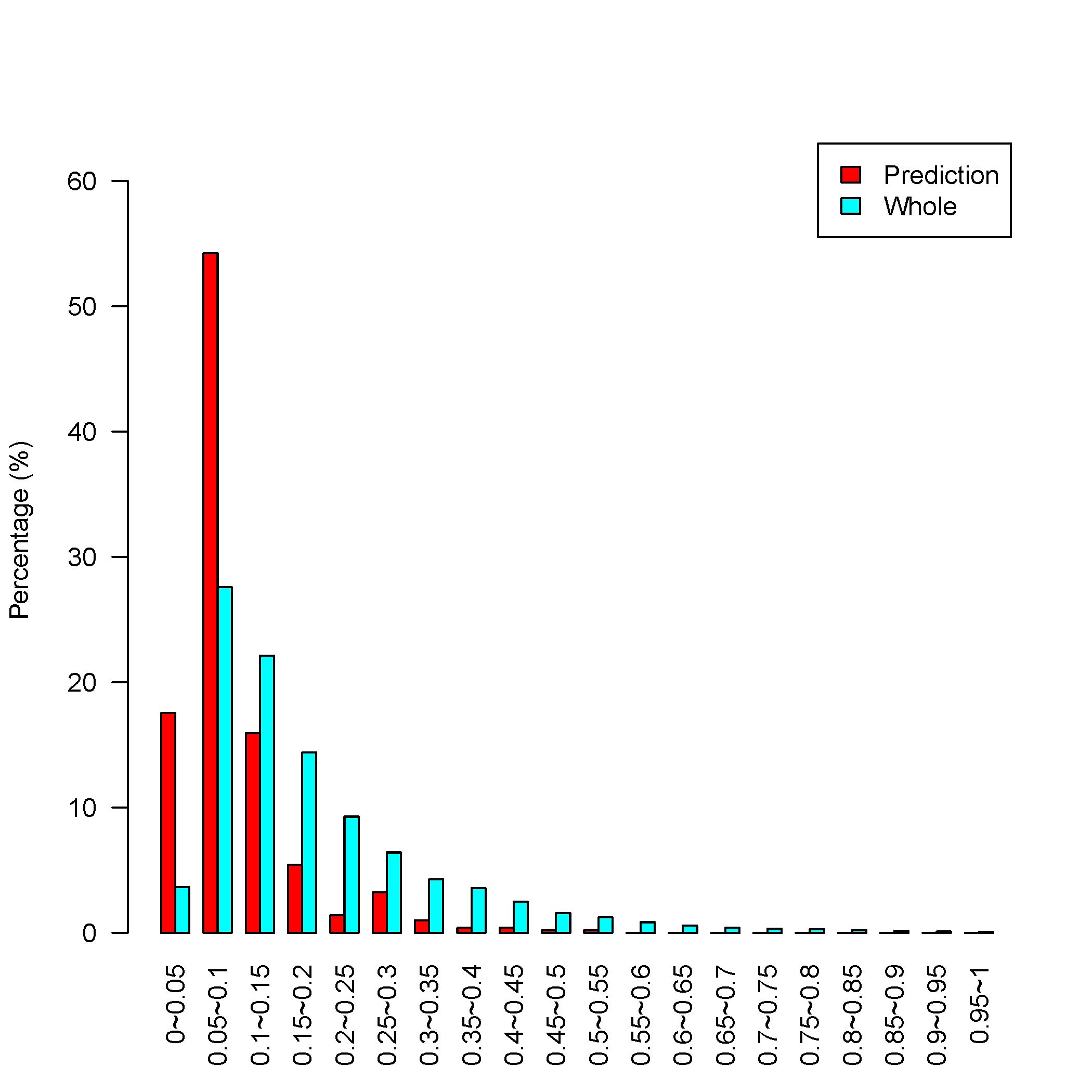


4. The details about the simulation

We constructed 4 groups of artificial time series, each has 50 time points.

A: 1 power unit pure Gauss white noise, representing HKGs. According to the definition of an HKG, we have assumed that the expression level of an HKG should be a constant to which is added the Gauss white noise (the latter caused by the inevitable factors of the experiments). However, our original Hela cell expression data had been normalized. Thus, the constant component of the time series had been removed, leaving only the Gauss white noise.

B: 5 power unit pure Gauss white noise, representing genes with no significant expression patterns. This group is designed for testing the robustness of the method, because the structure of the frequency spectrum of time series in this group is somewhat similar with the ones in the group A.

C: 4 power unit cosine wave with 3 cycles over the whole length and random phases adding 1 power unit Gauss white noise, representing genes having one dominating frequency component.

D: Superposition of two 4 power unit cosine waves with 3 and 7 cycles and random phases adding 1 power unit Gauss white noise, representing genes having two dominating frequency components.

Each group has 400 time series. We tried to distinguish every group from the others with SVM.

When one group is to be distinguished, 300 time series of which were chosen and defined to be the positive part of the SVM model. The rest 100 time series of this group were to be identified and were merged to the “putative” set.

Then we chose 300 time series from the rest 3 groups, 100 each. These 300 time series were merged to the “putative” set. All the 900 rest time series of the 3 groups were merged together and defined to be the “negative set”.

Then we did the supervised learning and testing, checking whether the SVM model could recognize the 100 series from the same group out of 400 total “putative” series. Every time we did the learning, we randomly chose 300 time series from the “negative set” and defined them to be the negative part of the SVM model.

We did the modeling and testing 1024 times. The accuracies were listed on the table:

When identifying time series from group A, RBF Kernel C=0.5 r=0.4

| A group identified | B group  miss identified | C group  miss identified | D group  miss identified |
| --- | --- | --- | --- |
| 100% | 11% | 0 | 0 |

When identifying time series from group B, RBF Kernel C=0.5 r=0.4

| A group miss identified | B group identified | C group  miss identified | D group  miss identified |
| --- | --- | --- | --- |
| 0 | 96% | 0 | 0 |

When identifying time series from group C, RBF Kernel C=0.5 r=0.4

| A group miss identified | B group miss identified | C group identified | D group  miss identified |
| --- | --- | --- | --- |
| 0 | 0 | 100% | 0 |

When identifying time series from group D, RBF Kernel C=0.5 r=0.4

| A group miss identified | B group miss identified | C group miss identified | D group identified |
| --- | --- | --- | --- |
| 0 | 0 | 0 | 100% |
